# Evaluation of Influence of Acupuncture and Electro-Acupuncture for Blood Perfusion of Stomach by Laser Doppler Blood Perfusion Imaging

**DOI:** 10.1093/ecam/nep050

**Published:** 2010-10-20

**Authors:** Zhang Dong, Li Shun-Yue, Wang Shu-You, Ma Hui-Min

**Affiliations:** Department of Biomedical Engineering, Institute of Acupuncture and Moxibustion, China Academy of Chinese Medical Sciences, Beijing 100700, China

## Abstract

The objective of this study is to observe effects of acupuncture and electro-acupuncture (EA) on blood perfusion in the stomach, and probe into the application of laser Doppler blood perfusion imaging technique in the study of the effect of acupuncture and moxibustion on the entrails. In the acupuncture group of 20 rats, acupuncture was given at “Zusanli” (ST 36) and in EA group of 18 rats, EA was applied at “Zusanli” (ST 36), with 18 rats without acupuncture used as control group. Changes of blood perfusion and microcirculation distribution in the stomach were investigated with laser Doppler blood perfusion imager (LDPI). The laser Doppler blood perfusion image could clearly display changes of blood flow distribution in the stomach before and after acupuncture. After acupuncture or EA was given at “Zusanli” (ST 36), the blood perfusion in the stomach increased significantly, the blood perfusion in the blood vessels and microcirculation of other parts significantly increased, and the maximum increase of the blood perfusion was found at 10 min after acupuncture or EA, with increases of 0.50 _ 0.11 (PU) and 0.66 _ 0.16 (PU), respectively, and the blood perfusion still kept at a higher degree within 10 min after ceasing of the acupuncture or EA. While the blood perfusion in the stomach in the rat of the control group tended to gradual decrease. It has been concluded that both acupuncture and EA can increase blood perfusion in the stomach, the EA having stronger action, and LDPI can display the regulative action of acupuncture on the blood vessel of the stomach by using an image.

## 1. Introduction

Acupuncture-moxibustion is a natural therapy and has very wide regulative action on the organism [1–8]. Because functions of some important entrails are often directly correlated with their blood perfusion, measurement of blood perfusion of tissues and organs after acupuncture and moxibustion is an important aspect for judging effects of acupuncture and moxibustion. Effects of acupuncture and moxibustion on the gastrointestinal tract have been studied more widely [9–11]. From the acupuncture efficacy of view, it is evidenced that increased blood perfusion flux occurs on correlative area with acupuncture [12]. However, general blood perfusion in the stomach after acupuncture and moxibustion had not been studied yet, because there was no ideal detective technique for measurement of general blood perfusion of the stomach. Laser Doppler blood image (LDPI) is a new blood perfusion imaging method and can display dynamically blood flow distribution on a wide range with an objective image [13, 14]. It cannot only measure microcirculation of the skin but also measure general blood perfusion of the internal organs. Previously, laser Doppler blood flow imaging technique has been used to observe blood perfusion of the abdominal organs, such as liver, spleen, kidney, urinary bladder, stomach, intestinal tract and mesentery [15]. In the present article, in order to investigate effects of acupuncture and moxibustion on blood perfusion in the stomach, laser Doppler blood flow imager was used for scanning and display of the stomach before and after acupuncture and electro-acupuncture (EA) at “Zusanli" (ST 36), which are reported in the following section.

## 2. Methods

### 2.1. Experimental Instruments

PeriScan PIM II laser Doppler blood perfusion imager (made by PERIMED Company, Sweden), laser wave length of 670 nm, was used in this study, with NR scanning pattern, Min scanning accuracy, about 20 cm of the distance from the scanning head to the detected object, and the image resource point of the picture usually used 30 (width) × 30 (highness), and image resource area 0.5 × 0.5 mm^2^. The instrument was linked with a computer and LDP12.5 imaging software was used for recording, save, analysis and processing of the blood perfusion image of gastric surface. In this study, the instrument system used has the functions of simultaneously recording both the laser blood perfusion image and the digital coded brightness image of the stomach (actual positional image of the examined part). The two images were used for control, so as to analyze the corresponding relation between the distribution of blood flow and the actual gastric surface position.

### 2.2. Experimental Animals

Fifty-six healthy adult Wistar rats, weighing 350 ± 50 g, either male or female, were used in the study, with 20 rats in the acupuncture group, 18 in the EA group and 18 in the control group. All experimental procedures were approved by the Ethical Committee of Academy of Medical Sciences, and conducted in accordance with the international accepted principles for laboratory animal use and care.

### 2.3. Experimental Environment

Experiments were carried out in an experimental box with a temperature of 30–32°C and a humidity of 80%–90%, the change of temperature before and after experiment for each rat was controlled within ±0.5°C.

The mean body temperature of the rats was 38.75±0.07°C (mean ± SE) in the acupuncture group, 38.80±0.06°C in the EA group and 38.70±0.06°C in the control group.

### 2.4. Preparation of *In Vivo* Rat Gastric Model

Water and food were suspended for the rats 24 h before preparation of the model. The rat was anesthetized by intraperitoneal injection of 2% pentobarbital sodium (2.5 mg/kg), and then a longitudinal incision below the xiphoid process was made along the ventral median line, and the gastric tissue was exposed.

### 2.5. Methods of Acupuncture and the Control Group

**Figure 1:**
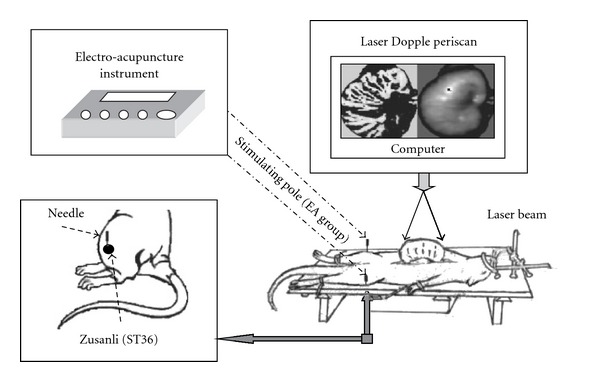
The gastric test method of LDPI, and acupuncture and EA method.

In the acupuncture group, acupuncture with 32^#^ needle of acupuncture was given at bilateral “Zusanli" (ST 36). After the needle was inserted, it was manipulated for one min and retained for 10 min. In the EA group, acupuncture with 32^#^ needle was given at bilateral “Zusanli" (ST 36) and it was manipulated for one min. Then they were connected with the stimulation electrodes of MBT-1 EA instrument (made by HUAYIN Ele. Inc., Changzhou, China) for stimulation of 10 min. The wave form was pulse wave, the duration of wave was 60 ms, the frequency was 1 Hz and the output power was 6 V stimulating voltage (Figure 1). In the control group, the natural fluctuation of the gastric blood flow was observed within 30 min.

### 2.6. Observation and Recording of Laser Doppler Blood Perfusion Image

The rat was placed in the box with constant temperature and the stomach was plainly spread on a background pad for laser examination of abdomen, and was placed directly below the laser scanner of the laser Doppler blood perfusion imager. Scanning model and imaging range were selected, and then the blood perfusion image of the observed part was recorded and saved in the computer for analysis (Figure 1).

Recording of effects of acupuncture or EA on blood flow in the stomach with Laser Doppler blood perfusion imaging technique: the control blood flow image of the stomach was recorded before acupuncture or EA, and then acupuncture or EA was given at bilateral “Zusanli” (ST 36) and the blood flow image of the stomach was recorded once every other 5 min, namely, the image was recorded once each at the instant of acupuncture or EA, acupuncture or EA for 5 min and 10 min, and 5 min and 10 min after ceasing the needling.

Recording of laser Doppler blood perfusion image of the stomach in the control group: the blood perfusion image was recorded five times, once every 5 min.

### 2.7. Analytical Methods for Laser Doppler Blood Perfusion Image

The LDP12.5 image software outfitted with this instrument was used for storing and saving of the blood perfusion image in the computer, and taking and analysis of relative data. The distribution of blood flow on different parts of the gastric surface was analyzed by laser Doppler blood perfusion image on the various parts of the stomach. Supposing the region of interest (ROI) in the midpoint of the stomach was round, 10 sites were selected for measurement of the blood perfusion in stomach. The value of measurement was figured by the perfusion unit in PERIMED instrument (perfusion units = CMBC (the concentration of measuring the volume inside the blood cells) ×*V* (the average velocity of blood cells), PU for short). The blood perfusions in the acupuncture group, EA group and the control group were measured and analyzed statistically, and the mean (of blood perfusion)—time curve diagram were delivered.

### 2.8. Statistical Analysis

The data were expressed as mean ± standard error ($\overset{¯}{X}\pm \text{SE}$). SPSS 13.0 statistical software was used and ANOVA test was adopted for comparisons between groups and *P<.05* was regarded as significant difference. The mean (of blood perfusion) time table and curve diagram were delivered.

## 3. Results

### 3.1. Manifestations of Laser Doppler Blood Perfusion Image of the Stomach after Acupuncture and EA

**Figure 2:**
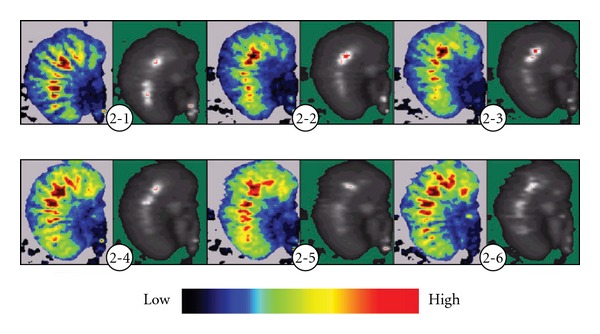
Continuous display of gastric laser Doppler blood perfusion image of rats during Acupuncture. Before acupuncture, most gastric blood flow were displayed as blue and dark green on the image, the blood vessels did not break, with less blood perfusion in the periphery vessels. After acupuncture, the light green and yellow regions increased gradually, indicating that blood perfusion the moderate and small vessels increase. At 5 and 10 min of acupuncture, blood perfusion of the microcirculation increase too. After ceasing of acupuncture, the light green regions increase whole, the yellow and red ranges extended further, indicating that acupuncture induces more obvious vasodilation in the stomach. (2-1) Before acupuncture; (2-2) instant of acupuncture; (2-3) acupuncture for 5 min; (2-4) acupuncture for 10 min; (2-5) 5 min after ceasing acupuncture; (2-6) 10 min after ceasing acupuncture.

**Figure 3:**
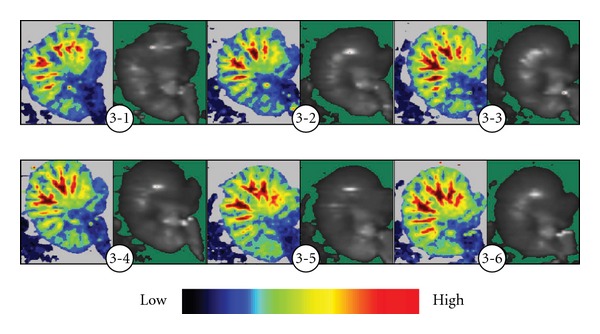
Continuous display of gastric laser Doppler blood perfusion image of rats during EA. Before EA, most gastric blood flow showed as blue and dark green on the image, with the large blood vessels showed shorter in yellow-red. After EA, the light green and yellow-red regions increased gradually, indicating that blood perfusion of the small vessels and microcirculation increased in dissimilar degrees. At 5 and 10 min of EA, the micro-vessels prolongated on the image. After ceasing of EA, the light green, yellow and red ranges extended further, indicating that EA induces more obvious dilation of vessels in the stomach. (3-1) Before EA; (3-2) instant of EA; (3-3) EA for 5 min; (3-4) EA for 10 min; (3-5) 5 min after ceasing EA; (3-6) 10 min after ceasing EA.

At the instant of acupuncture or EA at “Zusanli" (ST 36), the blood flow did not significantly increase. After 5 min of acupuncture or EA, the blood perfusion in the moderate and small blood vessels of the stomach increased significantly, with an increase of blood perfusion in the whole stomach. In addition, the blood perfusion of microcirculation in other parts of the stomach increased in dissimilar degrees, it more obviously increased in the parts close to the moderate and small blood vessels. At 10 min of acupuncture or EA the change of blood flow reached the largest, and it still kept at a higher level within 10 min after ceasing of acupuncture (Figures 2 and 3), indicating that acupuncture or EA has aftereffect, the acupuncture group and EA group have same action in increasing the gastric blood flow.

### 3.2. Displaying of the Control Group in Blood Perfusion Image of the Stomach

**Figure 4:**
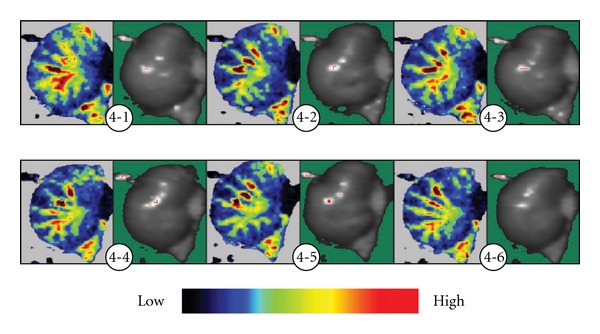
Continuous display of gastric laser Doppler blood perfusion image in rats of the control group. In the control group, the blood vessels showed radical distribution, and the blood vessel net displayed in green, yellow and red. In the period of observation, red and yellow decreased, blood perfusion in the end of the blood vessels and the periphery blood vessels decreased with poor filling degrees, and the blood perfusion did not increase until the end of observation of 25 min. (4-1) At 0 min; (4-2) at 5 min; (4-3) at 10 min; (4-4) at 15 min; (4-5) at 20 min; (4-6) at 25 min.

During the observation of 25 min, in the control group of 18 cases, gastric blood flow tended to gradually decrease in 10 cases, accounting for 66.7%, with more decrease of the blood perfusion in the vessels of micro-circulation than in moderate and small vessels (Figure 4). However, in the acupuncture or EA group, the blood perfusion in the moderate and small vessels and the micro-circulation increased simultaneously. In the control group, blood perfusion increased only in two cases, accounting for 13.3%, and did not significantly change in other three cases.

### 3.3. Statistical Analysis of Changes of the Mean Perfusion in the Acupuncture Group, the EA Group and the Control Group

**Figure 5:**
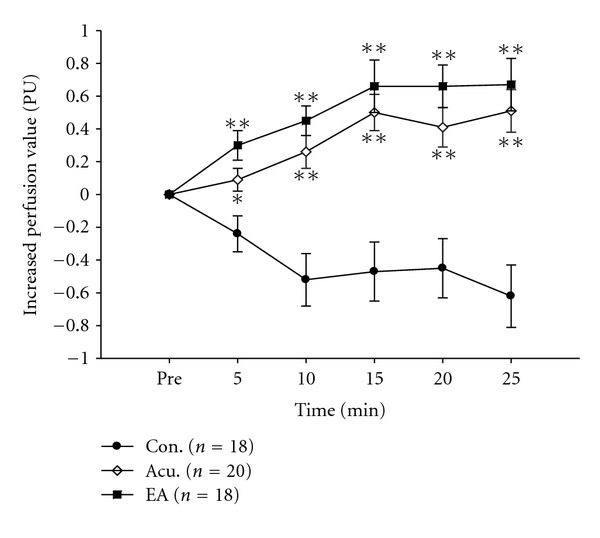
Changes of gastric perfusion in the three groups. (i) In acupuncture or EA group, 5 min is acupuncturing or EA instant, 10 min is 5 min, 15 min is 10 min of Acu. or EA 20 min is 5 min, 25 min is 10 min after ceasing Acu. or EA. (ii) Compared with the control group, *P < .002, **P < .001. (iii) There are not statistical differentia between acupuncture and EA group in all times.

Statistical analysis of mean perfusion indicated that the gastric blood perfusion tended to gradually decrease, the most decrease was found at 25 min, which was −0.62 ± 0.19 (PU) in the control group. After stimulation, the blood perfusion increased in both the acupuncture group and the EA group, the most increase was found at 10 min of stimulation, which were 0.50 ± 0.11 (PU) and 0.66 ± 0.16 (PU), respectively. After ceasing acupuncture or EA, blood perfusion did not continuously increase, but within 10 min after ceasing the stimulation, the blood perfusion still kept at a higher degree (Figure 5). Figure 5 and Table 1 show that there were statistically significant differences in the mean perfusion from that before acupuncture at all investigating time points as the acupuncture group and EA group compared with the control group (*P* < .001 or *P* < .002).

## 4. Discussion

The effect of acupuncture and moxibustion on the gastrointestinal tract is one of its important functions. Sun and Popova found that acupuncture could cure gastric ulcer and promote ulcerative union [16, 17]. Gao used acupuncture to treat atrophic gastritis [18], and Park to treat bacillary dysentery [19], attaining better therapeutic effects. Acupuncture and moxibustion achieved definite results in the study on alleviating injury of gastric mucosa [20]. The above actions of acupuncture and moxibustion are carried out possibly through improving gastric blood circulation, increasing nutrition, metabolism and anti-inflammatory substances in the lesion parts of the stomach. In the present article, the significant increase of gastric blood perfusion after acupuncture can provide experimental basis for explaining the mechanism of acupuncture and moxibustion for treatment of gastric diseases. Also, it was found that acupuncture and moxibustion had obviously regulative action on gastric myoelectric activity [21, 22], which was simultaneous with its regulative action on gastrointestinal peristalsis [23]. These findings and the increase of blood perfusion after acupuncture or EA found in the present article indicate that the regulative action on smooth muscle of the stomach and strengthening function of blood vessels are mechanisms of acupuncture and moxibustion in improving gastric functions and treating some lesion of the stomach.

It could be seen from the blood perfusion image of the stomach in this study that because distribution of gastric blood vessels showed radical characteristic, blood perfusion can be clearly differentiated and blood flow distribution state of the stomach can be seen at a glance. Based on previous our investigation, and characteristics and requirements of LDPI, the stomach was placed at the exterior of the body, with a lower temperature of the surface of the stomach, so at the natural state (control group), the gastric blood flow tended to reduction. After acupuncture or EA, obviously a potent vasodilator reaction of gastric vessels was induced, with blood perfusion in the moderate and small vessels and microcirculation increased in dissimilar degrees, and this increase is the result offsetting the natural decrease of blood flow, so the action of acupuncture and EA increasing blood flow is obvious. After ceasing acupuncture, the blood perfusion still increased as compared with that before acupuncture, indicating that acupuncture and EA has after-effect. After acupuncture or EA, the increase of blood flow in the moderate and small vessels and micro-vessels indicates that acupuncture or EA can strengthen the whole gastric blood perfusion.

In addition, another aim of the present experiment is understanding measure of blood flow of the stomach with laser Doppler blood perfusion imaging technique and evaluating effects of acupuncture and EA. It can be seen from the results in the experiment that changes of blood perfusion of the stomach and effects of acupuncture can be showed by LDPI, but cannot be reflected by other methods. Ultrasound Doppler's method, angiography, X-ray and CT, MR and other methods are difficulty used for direct measurement. The microsphere method is a new indirect measurement method of blood flow. It needs blood or tissue sample for measuring, the timing of test is limited; therefore the display of result is not as good as LDPI. Therefore, laser Doppler blood perfusion imaging technique makes investigation of blood flow state of the whole stomach and using it to study changes of vascular functions become possible. Laser Doppler blood perfusion flowmetry (LDPF) can detect blood perfusion of gastric mucosa at a fixed point, and it has been applied to evaluate efficacy of surgery operation [24]. Because laser beam is single and detective part is limited for LDPF, it cannot completely understand general blood flow state of the stomach and its changes. Laser Doppler blood perfusion imaging (LDPI) technique is a method which forms blood perfusion image via detecting laser beam on the basis of LDPF. Compared with LDPF, LDPI has these advantages: a wide range of detection, the beam shaping optics of scanner do not touch the detected entrails, able to imaging and quantificationally investigate vascular response at instant and in long-term course. Along with the requirement for blood flow detection in a large area of an organ, application of laser Doppler blood perfusion technique will have more important role.

Because the entrails are exposed at the exterior of the body, it needs a better environment of temperature and humidity for keeping physiologic function. In this experiment, the experimental box with constant temperature and humidity was used; making the stomach exposed at the exterior of the body still can keep at an approximate physiologic environment, with stable experimental results. In our laboratory, on the basis of more careful investigation on blood perfusion in many entrails by LDPI, the present study on effects of acupuncture and EA for whole gastric blood perfusion achieved ideal results for the first time. This possibly has a certain promoting action for application of this technique in acupuncture and moxibustion studies, and provides experimental data for understanding responses of the stomach in acupuncture and moxibustion, and explaining the mechanisms of acupuncture and moxibustion.

## Funding

Grants no. 30572418 from The National Natural Sciences Foundation of China.

## Figures and Tables

**Table 1:** Mean of gastric perfusion value in the three groups (mean ± SE, PU).

Group	*n*	Pre	5 min	10 min	15 min	20 min	25 min
Control	18	2.11 ± 0.18	1.87 ± 0.15	1.59 ± 0.15	1.64 ± 0.18	1.66 ± 0.19	1.49 ± 0.18
Acu.	20	2.14 ± 0.16	2.23 ± 0.13*	2.40 ± 0.13**	2.64 ± 0.17**	2.55 ± 0.16**	2.65 ± 0.17**
EA	18	2.08 ± 0.14	2.39 ± 0.15**	2.53 ± 0.15**	2.74 ± 0.23**	2.74 ± 0.19**	2.75 ± 0.22**

In acupuncture or EA group, 5 min is acupuncturing or EA instant, 10 min is 5 min, 15 min is 10 min of Acu. or EA. 20 min is 5 min, 25 min is 10 min after ceasing Acu. or EA. Compared with the control group, *P < .002, **P < .001. There are not statistical differentia between Acupuncture and EA group in all times.

## References

[1] Wang SY (2000). Experimental progress on acupuncture regulation of vesical function. *Shanghai Journal of Acupuncture and Moxibustion*.

[2] Li FH, Yang YG (1998). Research review on function regulation of gallbladder by acupuncture. *Shandong Journal of Traditional Chinese Medicine*.

[3] Zhao JX (2000). Progress in research of the function regulation of urinary system by the treatment of acupuncture. *Journal of Chinese Acupuncture and Moxibustion*.

[4] Long DH, He L, Hu L (2008). Evolvement of studies on mechanisms of acupuncture and moxibustion in improvement of myocardial ischemia. *Journal of Emergency in Traditional Chinese Medicine*.

[5] Ren QS, Wang JL (2008). Advancement in mechanism study of acupuncture treating ischemia cerebral injury. *Journal of Zhejiang University of Traditional Chinese Medicine*.

[6] Chen JS, Chen WK (2006). Mechanism of immunoregulation in therapy of acupuncture and moxibustion. *Liaoning Journal of Traditional Chinese Medicine*.

[7] Uchida S, Hotta H (2008). Acupuncture affects regional blood flow in various organs. *Evidence-Based Complementary and Alternative Medicine*.

[8] Inoue M, Kitakoji H, Yano T, Ishizaki N, Itoi M, Katsumi Y (2008). Acupuncture treatment for low back pain and lower limb symptoms—the relation between acupuncture or electroacupuncture stimulation and sciatic nerve blood flow. *Evidence-Based Complementary and Alternative Medicine*.

[9] Zhao H, Lai XS, Lian ZC (1998). Progress in research of regulation of stomach by acupuncture. *Journal of Guangzhou University of Traditional Chinese Medicine*.

[10] Ren TT (2006). Review in acupuncture effects of digestive hormone. *Journal of Liaoning University of Traditional Chinese Medicine*.

[11] Noguchi E (2008). Mechanism of reflex regulation of the gastroduodenal function by acupuncture. *Evidence-Based Complementary and Alternative Medicine*.

[12] Ng EYK, Goh CT, Wong PJ (2006). Evaluation of acupuncture weight loss programme using laser Doppler perfusion imaging. *Journal of Mechanics in Medicine and Biology*.

[13] Ng EYK, Fok SC, Goh CT (2003). Case studies of laser Doppler imaging system for clinical diagnosis applications and management. *Journal of Medical Engineering and Technology*.

[14] Movahed P, Evilevitch V, Andersson TLG (2005). Vascular effects of anandamide and N-acylvanillylamines in the human forearm and skin microcirculation. *British Journal of Pharmacology*.

[15] Zhang D, Wang S, Li S, Ma H (2006). Display of laser doppler perfusion imaging(LDPI) for entrails. *Journal of Chinese Microcirculation*.

[16] Sun JP, Pei HT, Jin XL, Yin L, Tian QH, Tian SJ (2005). Effects of acupuncturing Tsusanli (ST36) on expression of nitric oxide synthase in hypothalamus and adrenal gland in rats with cold stress ulcer. *World Journal of Gastroenterology*.

[17] Popova SP (2002). Effect of acupuncture on dynamics of changes in lipid peroxidation and activity of antioxidant system in rats with gastroduodenal ulcer. *Fiziolohichnyi Zhurnal*.

[18] Gao X, Rao H, Wang Y, Meng D, Wei Y (2005). Protective action of acupuncture and moxibustion on gastric mucosa in model rats with chronic atrophic gastritis. *Journal of Traditional Chinese Medicine*.

[19] Park ES, Jo S, Seong JK (2003). Effect of acupuncture in the treatment of young pigs with induced Escherichia coli diarrhea. *Journal of Veterinary Science*.

[20] Freire AO, Sugai GCM, Blanco MM, Tabosa A, Yamamura Y, Mello LEAM (2005). Effect of moxibustion at acupoints Ren-12 (Zhongwan), St-25 (Tianshu), and St-36 (Zuzanli) in the prevention of gastric lesions induced by indomethacin in Wistar rats. *Digestive Diseases and Sciences*.

[21] Shiotani A, Tatewaki M, Hoshino E, Takahashi T (2004). Effects of electroacupuncture on gastric myoelectrical activity in healthy humans. *Neurogastroenterology and Motility*.

[22] Liu J-H, Yan J, Yi S-X, Chang X-R, Lin Y-P, Hu J-M (2004). Effects of electroacupuncture on gastric myoelectric activity and substance P in the dorsal vagal complex of rats. *Neuroscience Letters*.

[23] Tabosa A, Yamamura Y, Forno ER, Mello LEAM (2004). A comparative study of the effects of electroacupuncture and moxibustion in the gastrointestinal motility of the rat. *Digestive Diseases and Sciences*.

[24] Monnet E, Pelsue D, Macphail C (2006). Evaluation of laser doppler flowmetry for measurement of capillary blood flow in the stomach wall of dogs during gastric dilatation-volvulus. *Veterinary Surgery*.

